# Midline sacral meningeal cysts: Neurophysiology abnormalities and their correlation with pelvic sensory and visceral symptoms

**DOI:** 10.1111/ene.16530

**Published:** 2024-11-05

**Authors:** Ivan Cabrilo, Claire Hentzen, Prasad Malladi, Sara Simeoni, Gérard Amarenco, Nathalie Zaidman, Mahreen Pakzad, Sachit Shah, Adrian T. Casey, Jalesh N. Panicker

**Affiliations:** ^1^ Victor Horsley Department of Neurosurgery The National Hospital for Neurology and Neurosurgery London UK; ^2^ Spinal Unit Wellington Hospital London UK; ^3^ Department of Neurosurgery Neurocentre of Southern Switzerland, Ente Ospedaliero Cantonale Lugano Switzerland; ^4^ Department of Uro‐Neurology The National Hospital for Neurology and Neurosurgery London UK; ^5^ GRC 01, GREEN Groupe de Recherche Clinique en Neuro‐Urologie AP‐HP, Sorbonne Université Paris France; ^6^ Faculty of Brain Sciences Queen Square Institute of Neurology, University College London London UK; ^7^ Lysholm Department of Neuroradiology The National Hospital for Neurology and Neurosurgery London UK

**Keywords:** lower urinary tract symptoms, meningeal cyst, pelvic neurophysiology, sacrum, spinal nerve roots

## Abstract

**Background and purpose:**

Midline sacral meningeal cysts (MSMCs) are cerebrospinal fluid‐filled dural diverticula. Although widely considered asymptomatic, cases involving voiding difficulties or pain have been reported. The aims of this study were, firstly, to describe the clinical presentation of patients with symptomatic MSMCs, secondly, to assess the impact of the cyst on nerve root function, and, thirdly, to assess whether nerve root injury is more frequent in patients with MSMCs than those with Tarlov cysts (TCs).

**Methods:**

Consecutive patients with MSMCs presenting with at least one pelvic symptom participated in a cross‐sectional review of symptoms using validated questionnaires. Findings of pelvic neurophysiology (pudendal sensory evoked potentials, sacral dermatomal sensory evoked potentials, external anal sphincter electromyography) and urodynamic testing were collected retrospectively. The relationship between neurophysiology, magnetic resonance imaging findings and patients' symptoms were assessed using Fisher's and analysis of variance tests. Neurophysiology findings were compared with those of TC patients.

**Results:**

Eleven female patients were included (mean age 42.3 ± 12.4 years). All reported urinary symptoms. Back pain (91%), radicular leg pain (91%), bowel symptoms (45%) and sexual dysfunction (75%) were also frequently reported. Nine patients (82%) had abnormal findings on neurophysiology; three patients (27%) had one abnormal test, and six (55%) had two abnormal tests. Patients with MSMCs were more likely to have at least two abnormal neurophysiology test results compared to TC patients (55% vs. 18%, respectively; *p* = 0.018).

**Conclusion:**

Our results indicate that MSMCs are indeed associated with injury to the sacral somatic innervation when symptomatic. MSMCs are more likely to cause sacral nerve root damage compared to TCs.

## INTRODUCTION

Midline sacral meningeal cysts (MSMCs) are extradural outpouchings that form as diverticula deriving from the dural lining of the sacral thecal sac [1, 2]. They are in communication with the subarachnoid space of the lumbar cistern through a dural pedicle (ostium) and are therefore also filled with cerebrospinal fluid (CSF). In general practice, they tend to be confused with perineurial cysts (also called Tarlov cysts [TCs]) as they both have in common certain radiological features, such as a sacral localisation, their CSF content, and their tendency for bony erosion. They are, in fact, distinct pathoanatomical entities, with TCs being intradural and MSMCs extradural, and with MSMCs characterised by the absence of nerve root fibres in their wall or within their lumen, which is in contrast to TCs. For this reason, MSMCs are classified as a type Ib spinal meningeal cyst and TCs as type II [3, 4]. A further difference is that TCs are usually lateralised as they arise from the nerve root sheath, whereas MSMCs tend to be midline structures, freely expanding bilaterally and craniocaudally within the epidural space of the sacral spinal canal [2].

Midline sacral meningeal cysts are considered rare and sources reporting on their prevalence are scarce, partly because they are often mistaken for TCs in the literature. One retrospective radiological study in a population of 1268 individuals sets their prevalence at 0.8% [5]. MSMCs are therefore less frequent than TCs, the prevalence of which is estimated at 4% [6, 7]. Similarly to TCs, MSMCs tend to be overlooked in radiology reports as they are widely considered to be benign lesions without clinical significance. Nevertheless, reports of symptomatic MSMCs have been described, thought to be related to the sacral nerve root compression that they exert, thereby causing radicular pain and genito‐urinary symptoms. The Valsalva manoeuvre can exacerbate pain, presumably in relation to an increase in hydrostatic pressure within the cyst lumen. Finally, as is the case with TCs, insufficiency fractures of the thinned‐out sacrum have also been reported [4, 6]. It has been estimated that 20%–25% of patients with TCs experience symptoms [8, 9], but the rate of symptomatic MSMCs remains unknown.

Similarly to TCs, different management options have been proposed for MSMCs, with varying rates of success in terms of symptom improvement and cyst recurrence. Such strategies include conservative medical management, percutaneous cyst aspiration with or without fibrin glue injection, and open surgery with several techniques having been described [1, 2, 3, 4, 6, 10, 11]. However, the actual impact of MSMCs on sacral nerve root function remains unknown and an objective assessment of radicular injury associated with this type of cyst is still lacking.

We recently investigated the impact of TCs on sacral nerve root function in a large consecutive cohort of patients and have shown that, in contrast to current understanding, presumedly symptomatic TCs are in fact associated with demonstrable injury to the sacral somatic innervation [12]. The aims of this study were firstly to describe the clinical presentation of a cohort of patients with presumedly symptomatic MSMCs, secondly, to assess the impact of the cyst on nerve root function and, finally, to compare the impact on sacral nerve function between our MSMCs and TC cohorts, given that these two lesions are in fact pathoanatomically distinct.

## METHODS

The Queen Square Clinical Audit Committee approved this study as an audit (registration number: 15‐202122‐CA) with a waiver of informed consent because assessments were conducted as part of routine clinical management.

### Patient selection, cross‐sectional assessment and clinical examination

Consecutive patients with symptomatic MSMCs (i.e., with at least one pelvic symptom) referred for a uro‐neurology assessment between January 2017 and July 2021 were included. Patients' medical records were reviewed for past or present medical conditions that may confound their neurophysiology findings and, if present, these patients were excluded from the cohort.

Patients underwent a cross‐sectional neurological and pelvic organ evaluation by experienced uro‐neurologists (J.N.P., S.S.). Onset of symptoms and profile of sensory, bladder (stress incontinence, overactive bladder [OAB], voiding difficulties), bowel (constipation, faecal incontinence) and sexual symptoms (arousal or orgasmic disorder) that were systematically collected at the time of the clinic visit were obtained from a review of medical records. Bladder, sexual and bowel symptoms were objectively assessed using the following validated questionnaires: the Urinary Symptom Profile (USP) to assess OAB, stress urinary incontinence (SUI) and low stream symptoms [13], the Constipation Scoring System (CSS) [14] and the Arizona Sexual Experience Scale (ASEX) to identify sexual dysfunction [15].

Patients underwent a structured pelvic neurological examination that included an evaluation of dermatomal sensation using a Neurotip and Von Frey hairs, providing a semi‐objective assessment of tactile sensations, sacral reflexes and motor function of the external anal sphincter (EAS) [16].

### Neurophysiology testing

Pelvic neurophysiology (PN) studies (Sierra® Summit™, MedEvolve, Little Rock, AR, USA) were performed by a neurophysiology scientist (P.M.) to evaluate the integrity of the sacral somatic sensory (pudendal, and S2 and S3 dermatomal sensory evoked potentials [SEPs]) and motor innervation (EAS electromyography). Data were collected directly on the machine, and the position of the markers was checked if the values were outside the limits. Technical aspects of the tests and criteria for abnormalities have been described in previous studies and are detailed in Table 1 [12, 17].

**TABLE 1:** Technical description of pelvic neurophysiology and criteria for abnormal findings.

Neurophysiology test	Technical description		Criteria for abnormal findings
EAS electromyography	Exploration of the anterior and posterior parts of the EAS bilaterally with a standard concentric needle 20 different motor unit potentials sampled on each side, with measure of duration, turns and area of recording		≥2/3 parameters abnormal (duration ≥13.7 ms; turns ≥6.3; area ≥1269 μVms)^a^
Pudendal somatosensory evoked potential	*Electrical stimulation* with silver/silver chloride 30 × 22 electrode size gel stickers over the clitoris at 3–4 times the sensory perception threshold at a rate of 3.1 Hz, 0.2 ms duration and within 50 mA	*Recording*: surface silver chloride disk electrodes over the scalp, 2 cm behind Cz (Cz‐2), overlying the sensorimotor cortex for the lower limbs and perineum, referred to Fpz (10–20 International System; filter setting 5–3000 Hz). Onset latency and peak‐to‐peak amplitude were measured	absent, or P40 latency >2 standard deviations (SD) from the mean (42.8 ms in women and 47.6 ms in men), or >50% inter‐side difference in amplitude^b^, ^c^
Dermatomal somatosensory evoked potential	*Electrical stimulation* with silver/silver chloride 30 × 22 electrode size gel stickers over the respective sacral dermatomes at 3–4 times the sensory perception threshold at a rate of 3.1 Hz, 0.2 ms duration and within 50 mA		absent, or inter‐side latency difference >4.5 ms, or >50% inter‐side difference in amplitude^d^, ^e^

Abbreviations: EAS, external anal sphincter; SD, standard deviation.

^a^ Podnar (2004) [18].

^b^ Pelliccioni et al. (2014) [19].

^c^ Ormeci et al. (2017) [20].

^d^ Storm and Kraft (2004) [21].

^e^ Dikmen and Oge (2013) [22].

Abnormal findings were defined [18, 19, 20, 21, 22] as stated in Table 1. Incidental abnormalities have not been reported in healthy women [23].

### Urodynamic studies

Urodynamic studies comprised non‐invasive uroflowmetry and measurement of postvoid residual urine volume (PVR), and invasive multichannel cystometrogram, including filling cystometry (medium fill at 50 mL/min) and pressure flow studies (Medical Measurement Systems) following International Continence Society Good Urodynamic Practices [24]. A PVR measurement of >100 mL on uroflowmetry was considered significant for urinary retention. The need for urodynamic assessment was left to the discretion of the physician and performed only in patients who were reporting significant lower urinary tract symptoms or significant voiding dysfunction. Urodynamic findings were reviewed by a urologist (M.H.P.) and a uro‐neurologist (C.H.) to look for evidence of detrusor overactivity, impaired bladder sensation, bladder outlet obstruction or detrusor underactivity [25, 26, 27].

### Magnetic resonance imaging

Magnetic resonance imaging (MRI) of the lumbosacral spine acquired closest to the date of the neurophysiology investigation was reviewed by the same observer (I.C.) and sacral cystic lesions other than MSMCs were excluded [2, 3]. In cases of radiological ambiguity with TCs, a second expert opinion was sought (A.T.C.). The following parameters were evaluated: (a) MSMC extension in terms of vertebral levels; (b) MSMC dimensions (greatest length in the craniocaudal, mediolateral and ventrodorsal axes, respectively); and (c) the nerve roots found to be compressed by the MSMC.

### Comparison with TC patients

We carried out an analogous assessment (neurophysiology testing, imaging review and pelvic symptom questionnaires) in a consecutive cohort of 65 female patients with symptomatic sacral TCs referred to our group during the same period of inclusion as the present study's MSMC patient cohort [12]. The two groups were studied together as part of the same study protocol and permissions from the Queen Square Clinical Audit Committee were the same, but as they were affected by different pathological entities, they were each the subject of a separate report. Given that the methodology was identical (same period of inclusion, same investigators for each step) for both studies, abnormal neurophysiology results could be compared between our TC [12] and MSMC patient cohorts.

### Statistical analysis

Analysis was performed in RStudio (Version 1.2.5033, RStudio: Integrated Development for R. RStudio, Inc.). Clinical and demographic characteristics, results of investigations and questionnaire data were summarised as frequency (per cent), mean (SD), or median (interquartile range [IQR]). Statistical analyses were limited due to the small number of patients and non‐parametric tests were used (analysis of variance tests). Fisher's test was used to compare qualitative parametric data between MSMC and TC patients. *p* values < 0.05 were taken to indicate statistical significance.

## RESULTS

Twelve patients with MSMCs were reviewed. The only male patient in the cohort also had a history of cancer with chemotherapy, raising the possibility of concomitant chemotherapy‐induced neuropathy, and was therefore excluded. Eleven patients, all female, were included for final analysis, with a mean age of 42.2 ± 12.5 years. Demographic characteristics and description of pelvic symptoms are reported in Table 2.

**TABLE 2:** Patient demographics and clinical presentation in 11 patients with midline sacral meningeal cyst.

Patient demographics and clinical presentation	
Patient characteristics	
Age, mean ± SD years	42.3 ± 12.4
Parity, median (IQR)	1 (0–2)
Duration of symptoms, median (IQR) years	4 (3–5)
Medication, *n* (%)	
Opioids	1 (9)
Other painkillers^a^	5 (45)
Symptoms	
Sensory symptoms, *n* (%)	
Back pain	10 (91)
Radicular pain (buttock/leg)	10 (91)
Pelvic pain	5 (45)
Numbness	4 (36)
Buttock/leg	4 (36)
Perineal area	1 (9)
Urinary symptoms, *n* (%)	
Self‐reported	11 (100)
Urgency	7 (64)
Frequency	7 (64)
Urgency urinary incontinence	6 (54)
Stress urinary incontinence	5 (45)
Voiding difficulties	8 (73)
USP score (*n* = 8), median (IQR)	
Stress urinary incontinence subscore	1.5 (0–4.25)
Overactive bladder subscore	10 (5–12.75)
Low stream subscore	3 (2.25–3)
Bowel symptoms (*n* = 10), *n* (%)	
Self‐reported	5 (50)
Constipation	2 (20)
Faecal urgency	3 (33)
Faecal incontinence	2 (22)
Constipation Scoring System (*n* = 6), median (IQR) score	8 (6.5–8.75)
Sexual symptoms, *n* (%)	
Sexual dysfunction (ASEX; *n* = 8)	6 (75)

*Note*: USP (min/max score: 0/21); stress urinary incontinence (min/max score: 0/9); low stream symptoms (min/max score: 0/9); Constipation Scoring System (min/max score: 0/30); ASEX (five‐item scale; a total score ≥19 of 30 or a score of 5 in any question or ≥4 in at least three questions suggests sexual dysfunction).

Abbreviations: ASEX, Arizona Sexual Experience Scale; IQR, interquartile range; SD, standard deviation; USP, urinary symptom profile.

^a^ Including pregabalin, gabapentin, duloxetine, amitriptyline.

### Neurophysiology findings

All patients underwent neurophysiology testing, and the median (IQR) interval between their closest MRI study and neurophysiology tests was 6 (3–14) months. Nine patients (82%) had abnormal findings on neurophysiology; three patients (27%) had one abnormal test, and six (55%) had two abnormal tests. There was no association between abnormal neurophysiology findings and age (*p* = 0.28) or parity (*p* = 0.66).

Seven patients had abnormal dermatomal SEPs (64%); five patients had abnormalities in one dermatome, two patients in three dermatomes. Seven patients had abnormal pudendal SEPs (64%), and six patients had unilateral abnormality, while one patient had bilateral pudendal SEP abnormalities. Only one patient (11%) had abnormal EAS electromyography.

For each patient, pelvic symptoms, and neurophysiology, imaging and uroflowmetry findings are detailed in Table 3.

**TABLE 3:** Clinical presentation and neurophysiology, magnetic resonance imaging and uroflowmetry findings in 11 patients with midline sacral meningeal cysts.

Patient	Age	Symptoms						Neurophysiology findings			MRI findings		Uroflowmetry	
		Pain	Numbness	LUTS storage	LUTS voiding	Bowel symptoms	Sexual dysfunction	EAS EMG	Dermatomal SEPs^a^ (*n*; level)	Pudendal SEPs	Extension of the cyst	Size of the cyst	Curve	PVR
1	54	Back Legs	Legs Perineum	Yes	No	No	NA	Normal	Abnormal (3; R‐S2, L‐S2, R‐S3)	Abnormal (bilateral)	S1 to S5	96 × 47 × 25	Normal	Normal
2	49	Back Legs	–	Yes	No	Yes	Yes	Normal	Abnormal (1; L‐S3)	Abnormal (unilateral)	S1 to S3	40 × 35 × 24	Normal	Normal
3	59	Legs Pelvis	–	Yes	Yes	No	Yes	Normal	Normal	Abnormal (unilateral)	S1 to S4	80 × 39 × 22	Abnormal	High
4^b^	25	Back Legs Pelvis	–	No	Yes	NA	No	Normal	Normal	Normal	S2 to S4	60 × 43 × 20	NA	Normal
5	58	Back Legs Pelvis	–	Yes	Yes	No	Yes	Not done	Abnormal (1; L‐S2)	Normal	S2	14 × 12 × 20	Abnormal	Normal
6	35	Back Legs	–	Yes	No	No	NA	Normal	Abnormal (3; R‐S2, R‐S3, L‐S3)	Abnormal (unilateral)	S2	19 × 11 × 19	Abnormal	High
7^b^	34	Back Legs	–	Yes	Yes	Yes	NA	Normal	Normal	Abnormal (unilateral)	S2 to S4	57 × 30 × 13	Normal	Normal
8	47	Back Pelvis	Leg	Yes	Yes	No	No	Abnormal	Abnormal (1; R‐S3)	Normal	S1 to S3	51 × 28 × 14	Normal	Normal
9	43	Back Legs	Leg	Yes	Yes	Yes	Yes	Normal	Abnormal (1; L‐S2)	Abnormal (unilateral)	S1 to S4	83 × 51 × 36	Normal	High
10	39	Back Legs Pelvis	‐	Yes	Yes	Yes	Yes	Not done	Normal	Normal	S2 to S4	57 × 39 × 21	Normal	Normal
11	22	Back Legs	Buttock	Yes	Yes	Yes	Yes	Normal	Abnormal (1; L‐S2)	Abnormal (unilateral)	S1 to S2	28 × 20 × 14	Abnormal	Normal

Abbreviations: EAS‐EMG, external anal sphincter electromyography; L, left; LUTS, lower urinary tract symptoms; MRI, magnetic resonance imaging; NA, not applicable (because not reported/not performed); PVR, postvoid residual urine volume; R, right; SEP, sensory evoked potential.

^a^ S2 and S3 dermatomal SEPs were explored. The number (*n*/4) and level (S2 and/or S3, left and/or right) of abnormal responses is indicated.

^b^ Other urological conditions (Fowler's syndrome).

### Association with MRI findings

The MRI results showed compression of a median of 6 (range 3–8) sacral nerve roots and an average of 6.4 nerve roots. No association was observed between abnormal dermatomal SEPs and compression of the related sacral nerve roots. Abnormal neurophysiology results did not seem to depend on the dimensional extent of the MSMC, as neither the number of sacral vertebral levels (*p* = 0.94) nor the greatest dimension in the mediolateral axis (*p* = 0.58) correlated with neurophysiology findings. Indeed, the two patients with the smallest lesions (Patients 5 and 6 in Table 3) both had abnormal neurophysiology findings (one and two abnormal tests, respectively).

### Association with pelvic symptoms and urodynamic findings

All patients reported lower urinary tract symptoms, with 91% reporting storage symptoms and 73% voiding difficulties. Ten patients underwent uroflowmetry, four of whom had an abnormal trace, and three of whom had a significant PVR.

Four patients underwent cystometrogram, all of whom presented abnormal findings. Three of these patients were found to have impaired bladder sensation (Patients 3, 6 and 7) and one patient had increased bladder sensation (Patient 10). Two patients demonstrated detrusor overactivity, one of whom had normal neurophysiology tests (Patient 10) and the other abnormal pudendal SEPs as well as features suggestive of Fowler's syndrome (Patient 7).

Bowel symptoms were initially reported by five patients, but among the seven patients who completed the CSS questionnaire, the minimum score was 5, indicating at least a mild degree of constipation in all responders.

Of the eight patients who completed the ASEX questionnaire, six (75%) had sexual dysfunction.

Statistical analyses based on pelvic symptoms were not performed due to the small number of patients.

### Comparison with TC patients

Sixty‐five female patients (mean age 51.2 ± 12.1 years) were included in our previous study assessing nerve root injury in the presence of sacral TC, using the same PN assessment [12]. Patients with TCs were significantly older than those with MSMCs (*p* = 0.03). The mean USP subscores and CSS scores were not different between the two populations (*p* > 0.05). The proportion of abnormal neurophysiology tests was not significantly different between TC and MSMC patients (57% vs. 82%, respectively; *p* = 0.19). Considering the number of abnormal tests, a significant difference was found between the two cohorts (*p* = 0.046), and the proportion of patients with at least two abnormal tests was significantly higher in patients with MSMCs than in those with sacral TCs (55% vs. 18%, respectively; *p* = 0.018 [Table 4]).

**TABLE 4:** Comparison of abnormal neurophysiology findings in patients with midline sacral meningeal cysts and sacral Tarlov cysts.^a^

	MSMCs (*n* = 11)	Sacral TCs^a^ (*n* = 65)	*p* value
Abnormal neurophysiology, *n* (%)			
Yes	9 (82)	37 (57)	0.19
No	2 (18)	28 (43)	
Number of abnormal tests, *n* (%)			
0	2 (18)	28 (43)	0.046
1	3 (27)	25 (39)	
2	6 (55)	10 (15)	
3	0	2 (3)	

Abbreviations: MSMC, midline sacral meningeal cyst; TC, Tarlov cyst.

^a^ Hentzen et al. [12].

## DISCUSSION

To the best of our knowledge, this is the first study to objectively assess the impact of MSMCs on sacral nerve root function. Nine of our 11‐patient symptomatic cohort had abnormal neurophysiology findings, most with at least two abnormal tests. Compared to TCs, sacral nerve injury was found to be more frequent and more pronounced with MSMCs. Association between pain and genito‐urinary symptoms is frequent and the high rate of nerve root injury makes it plausible that pelvic symptoms are occurring due to the cyst.

### Midline sacral meningeal cysts: A pathoanatomical entity distinct from TCs

**FIGURE 1:**
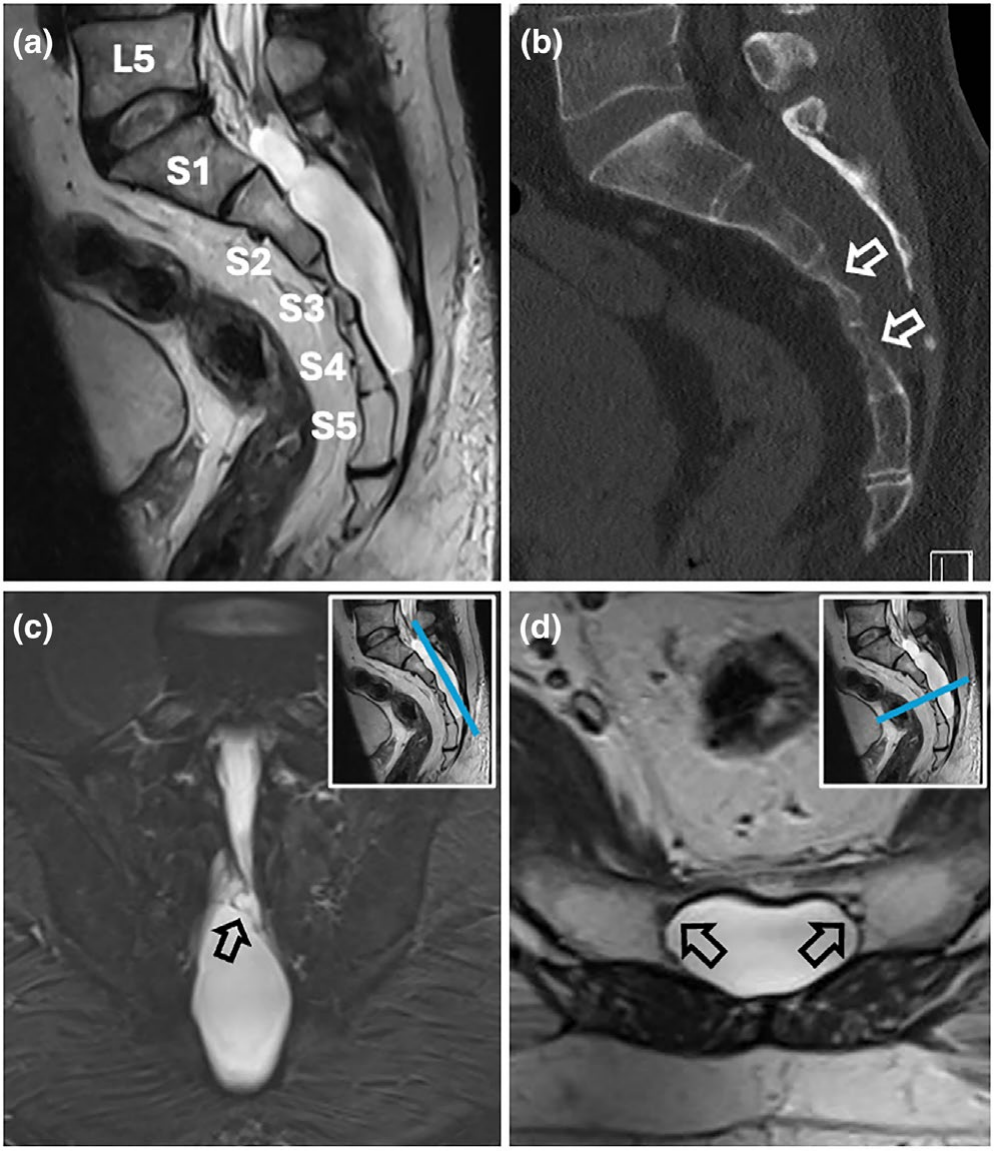
Imaging features of midline sacral meningeal cysts (MSMCs). (a) Sagittal T2‐weighted lumbosacral magnetic resonance imaging (MRI) slice showing an MSMC from lower S1 to the S4/S5 junction. (b) Sagittal lumbosacral computed tomography slice (bone window) demonstrating MSMC‐induced sacral scalloping (white arrows). (c) Oblique coronal T2‐weighted sacral MRI slice running through the MSMC's ostium (black arrow), seen to present with complex septations. Inset in right upper corner indicates level of the MRI slice. (d) Axial T2‐weighted sacral MRI slice through lower S3 level. Black arrows indicate S4 nerve roots pushed ventrally by the dorsally growing cyst.

Whilst TCs are dilatations of the perineurial space within nerve root sheaths, MSMCs are true dural diverticula, namely, epidural outpouchings with a dural lining (Figure 1) [2, 3]. They are expansive lesions within the sacral canal and, depending on whether they arise from the ventral or the dorsal aspect of the dural sac, will compress the sacral nerve roots against the dorsal or ventral surface of the sacral canal, respectively (Figure 1d) [11]. MSMC and TC interactions with the sacral nerve roots are therefore very different: in TCs, the nerve root fibres are either splayed out in the cyst's wall or run through the cyst's lumen [1, 2, 3]; in MSMCs, the sacral nerve roots are extrinsically compressed, as they would be with an epidural tumour. Moreover, whilst a TC is ‘confined’ to its one nerve root (with some mass effect nevertheless possible on neighbouring nerve roots [12]), an MSMC will typically compress multiple nerve roots. Indeed, in our MSMC cohort, MRI showed compression of a median of 6 sacral nerve roots. This may account for the higher number of neurophysiology abnormalities in MSMC patients compared to TC patients.

### Pelvic neurophysiology findings

Similar to our findings in TC patients [12], the neurophysiology abnormalities in MSMC patients preferentially involved sensory over motor pathways. We hypothesise that this is related to nerve fibre size, sensory nerves being thinner and less myelinated than motor nerve fibres, and therefore more sensitive to compression‐induced injury, along the same line of thought as that in nerve root compression in the far more frequent setting of spinal degenerative changes.

Despite an apparently greater prevalence of abnormal neurophysiology tests in MSMC compared to TC patients (82% vs. 57%), this difference was not found to be significant, although that may be related to a lack of power due to the small number of MSMC patients.

There are, to our knowledge, no other publications reporting on PN findings in patients with MSMCs with which to compare our results. Furthermore, only a few studies have reported neurophysiology findings in patients with sacral TCs. Cattaneo et al. [28] observed sural nerve conduction abnormalities that were consistent with the location of the cyst and support cyst involvement in nerve damage. Hulens et al. [29] registered ≥50% neurogenic motor unit potentials in S2–S4 territories, indicative of axonal injury. However, these criteria have not been validated for external anal sphincter testing and may lead to an overestimation of encountered anomalies. Similarly, the 40‐ms threshold their group chose when eliciting the anal reflex is lower than the norm set in the literature [30] and may explain the differences in the rates of reported motor anomalies in our respective cohorts.

### Association with pelvic symptoms and urodynamic findings

All patients described bladder symptoms, half complained of constipation and 75% of those who responded to the dedicated questionnaire reported sexual dysfunction. It is plausible that sacral nerve root injury may impact genito‐urinary functions as the sacral somatic and splanchnic innervation travel together in the sacral nerve roots [31]. Voiding difficulties would represent the most likely symptom, and a significant PVR or abnormal uroflowmetry would be expected findings [16]. Five patients had abnormal uroflowmetry, which may reflect the impact of nerve injury on bladder function. However, other factors such as medication use—particularly opiates—could also play a role.

The genito‐urinary system receives bilateral innervation from multiple (i.e., S2, S3 and S4) nerve roots; this redundance may play a role in the preservation of efficient voiding despite neurophysiologically demonstrated sacral nerve root injuries. Three patients had impaired bladder sensation during cystometry, which may be related to sacral nerve injury, but this would require further investigation to ascertain.

Although a high rate of storage symptoms was reported by our patients, OAB symptoms are in fact believed to be unrelated to sacral nerve injury. Such symptoms are common and various factors may be involved. Only four patients underwent cystometry, and the only patient found to have detrusor overactivity did not have neurophysiological abnormalities. Although the number of patients in our MSMC cohort is too small to reach a conclusion in this regard, these findings support the hypothesis that OAB and detrusor overactivity are in fact unrelated to MSMCs, as already shown in our TC patient cohort [12].

### Association with MRI findings

Our cohort was too small to identify a significant association between abnormal dermatomal SEPs and compression of the related sacral nerve roots. Also, the occurrence of abnormal neurophysiology results did not seem to depend on the size of the MSMC. This may indicate that, despite the characteristic expansiveness of MSMCs, typically causing multiple sacral nerve root compression on MRI, the additional requisite of raised intracystic pressure is necessary to cause actual nerve root injury.

Indeed, based on myelographic computed tomography and intraoperative findings, it has been suggested that a check‐valve mechanism, differentially trapping CSF within the cyst, may constitute the risk factor leading to a build‐up of intracystic pressure both in TCs and MSMCs [1, 2, 32, 33]. Compression‐induced neural injury and associated symptoms are thought to occur when the intracystic pressure threshold to induce neural injury has been reached. It has been ventured that intermittent Valsalva manoeuvres may precipitate symptoms by trapping CSF, past an efficient check‐valve mechanism, within the cyst lumen [2, 34]. Moreover, this model would also explain why an individual MSMC of only modest dimensions—but with a more efficient check‐valve mechanism—can be found to have caused more neurophysiological abnormalities than a much larger MSMC where an equilibrium between intracystic and perineurial pressures has been reached and that is below the necessary threshold to induce neural injury.

Accordingly, our group's treatment rationale for symptomatic MSMCs described in a previous publication [2] is to surgically access the lumen of the cyst through a sacral laminectomy to identify its ostium (Figure 1c) and close it off to prevent the cyst from recollecting.

### Study limitations

Due to the study's retrospective design, there was a time interval between symptom assessment and investigations such as neurophysiology, urodynamics and MRI. Therefore, a cross‐sectional assessment of current symptoms may not have captured symptoms related to changes in medications or progression of disease since the PN testing. Matching with a control cohort would have strengthened the study; however, the design of our study precluded this possibility as PN is not performed routinely in the absence of suspected nerve lesions.

The small number of patients is the main limitation of this study. The number of cystometrograms performed was too small to assess the impact of nerve injury on bladder contractility and sensation. Although PN techniques to assess the sacral innervation are well established, sacral dermatomal SEP studies are relatively new and are just being validated [35]. Nevertheless, this assessment is routinely performed at our centre by a trained scientist, effectively ensuring that technical issues were excluded.

### Future perspectives

Despite the small number of patients, the prevalence of abnormal neurophysiology findings was found to be high, and it is conceivable that these tests will advance the interpretation of symptoms in patients in whom the potential involvement of their MSMC is still widely excluded on the arbitrary assumption that these lesions are innocuous. Percutaneous needle aspiration of sacral cysts has been described as a means of distinguishing symptomatic cysts from asymptomatic ones, based on patient‐reported symptom relief following the procedure [36, 37]. However, it is of inconsistent interpretability, as an absence of symptom relief following cyst aspiration does not necessarily imply a lack of causality. Moreover, procedure‐related complications may also rarely occur [6, 38]; therefore, PN may represent a safe and non‐invasive alternative means to identify symptomatic MSMC.

The usefulness of PN as a screening method for MSMC involvement in patients with pelvic symptoms is, however, unclear. Indeed, it is likely that symptomatic MSMC‐induced nerve root compression need not necessarily be associated with neurophysiology anomalies, much in the same way that a symptomatic cervical or lumbar nerve root compression without objective neurological deficits frequently comes with a normal neurophysiology investigation of the upper and lower limbs, respectively [39, 40].

Abnormal PN findings will therefore be useful to identify, and select for surgery, those patients in an advanced stage of symptomatic MSMC, namely, those patients in whom neurophysiologically discernible—and possibly already even irreversible—neural injury has taken place. Such findings are very specific, so much so that the presence of even a single anomalous result on PN denotes sacral nerve root injury by the MSMC, thereby warranting active management. Normal PN findings should, however, not exclude MSMC involvement in a symptomatic patient in whom alternative urological, gynaecological or gastro‐intestinal aetiologies have been excluded. Needle aspiration of the cyst may therefore play a greater role in the diagnostic evaluation of patients with suspected MSMC‐related symptoms and in whom PN is normal. In addition to its diagnostic value in these cases, it can also be seen as an initial therapeutic attempt before surgery is considered in the event of symptom recurrence or persistence. As in other instances of symptomatic nerve root compression in the cervical or lumbar spine, in the absence of neurological deficits or neurophysiology anomalies, the decision to proceed with surgery or continue with conservative management is based on patients' tolerance of their symptoms.

In conclusion, this is the first study to report objective neurological impairment of sacral nerve root functions in patients with symptomatic MSMC and challenges the widespread assumption of the innocuousness of these lesions. Their expansiveness and the simultaneous involvement of multiple nerve roots may account for the more prevalent and pronounced nerve injuries compared with TCs. PN may represent a useful tool in the assessment of patients with MSMCs and may play a role in patient selection for surgery.

## AUTHOR CONTRIBUTIONS

**Ivan Cabrilo:** Conceptualization; data curation; formal analysis; investigation; methodology; visualization; writing – original draft; writing – review and editing. **Claire Hentzen:** Conceptualization; data curation; formal analysis; investigation; methodology; visualization; writing – original draft; writing – review and editing. **Prasad Malladi:** Data curation; investigation; methodology. **Sara Simeoni:** Conceptualization; validation. **Gérard Amarenco:** Conceptualization; validation. **Nathalie Zaidman:** Conceptualization; investigation. **Mahreen Pakzad:** Resources; validation. **Sachit Shah:** Conceptualization; methodology. **Adrian T. Casey:** Conceptualization; resources. **Jalesh N. Panicker:** Conceptualization; investigation; methodology; project administration; resources; supervision; writing – original draft; writing – review and editing.

## CONFLICT OF INTEREST STATEMENT

The authors have no conflicts of interest to declare.

## Data Availability

The data that support the findings of this study are available from the corresponding author upon reasonable request.
